# Carbon emissions from a temperate coastal peatland wildfire: contributions from natural plant communities and organic soils

**DOI:** 10.1186/s13021-021-00189-0

**Published:** 2021-09-01

**Authors:** Robert A. Mickler

**Affiliations:** Department of Forestry and Environmental Resources, North Carolina State University, 2820 Faucette Drive, Raleigh, NC 27695 USA

**Keywords:** C emissions, dNBR, Ground fire, LIDAR, Peatland, Temperate, Wildfire

## Abstract

**Background:**

One of the scientific challenges of understanding climate change has been determining the important drivers and metrics of global carbon (C) emissions and C cycling in tropical, subtropical, boreal, subarctic, and temperate peatlands. Peatlands account for 3% of global land cover, yet contain a major reservoir of 550 gigatons (Gt) of soil C, and serve as C sinks for 0.37 Gt of carbon dioxide (CO_2_) a year. In the United States, temperate peatlands are estimated to store 455 petagrams of C (PgC). There has been increasing interest in the role of wildfires in C cycling and altering peatlands from C sinks to major C sources. We estimated above- and below-ground C emissions from the Pains Bay Fire, a long-duration wildfire (112 days; 18,329 ha) that burned a coastal peatland in eastern North Carolina, USA.

**Results:**

Soil C emissions were estimated from pre- and post-burn Light Detection and Ranging (LIDAR) soil elevation data, soils series and C content mapping, remotely sensed soil burn severity, and post-burn field surveys of soil elevation. Total above-ground C emissions from the fire were 2,89,579 t C and 214 t C ha^−1^ for the 10 vegetation associations within the burn area perimeter. Above-ground sources of C emissions were comprised of litter (69,656 t C), shrub (1,68,983 t C), and foliage (50,940 t C). Total mean below-ground C emissions were 5,237,521 t C, and ranged from 2,630,529 to 8,287,900 t C, depending on organic matter content of different soil horizons within each of the 7 soil series. The mean below-ground C emissions within the burn area were 1,595.6 t C ha^−1^ and ranged from 629.3 to 2511.3 t C ha^−1^.

**Conclusions:**

In contrast to undisturbed temperate peatlands, human induced disturbances of the natural elevation gradient of the peatland has resulted in increased heterogeneity of floristic variation and assemblages that are a product of the spatial and temporal patterns of the water table level and the surface wetness across peatlands. Human induced changes in surface hydrology and land use influenced the fuel characteristics of natural vegetation and associated soils, thus influencing wildfire risk, behavior, and the resulting C emissions.

## Background

During the past 3 decades, a major scientific challenge of climate change has been determining the important drivers and metrics on C cycling in tropical, subtropical, boreal, subarctic, and temperate peatlands. Peatlands account for 3% of global land cover, yet contain major reservoirs of 550 gigatons of soil C (GtC), and serve as C sinks for 0.37 Gt of carbon dioxide (CO_2_) a year. These values account for an estimated 42% of global soil C stocks and the equivalent of 75% of all atmospheric C [1].

Brinson and Malvarez [2] identified five global regions with temperate peatlands: southern North America; northern Europe; northern Mediterranean; southern South America; southern Australia and New Zealand; and Russia, Mongolia, northeastern China, Korea, and Japan. Historic and current land use change within these five global regions has increased the uncertainty in quantifying the average long-term apparent rate of C accumulation (LORCA) in peatlands, the average recent rate of C accumulation (RERCA) in peatland distribution, and the spatial extent of peatland C. Relatively few studies have been conducted on peatlands in the temperate climate regions, despite the potential risk of wildfires and climate change altering their function and distribution [3–18]. The limited availability of temperate peatland data is especially true for continents dominated by boreal and tropical peatlands. Despite the paucity of LORCA and extent data, temperate peatlands are estimated to store 455 PgC [19], the equivalent of the C stored in all living fauna and flora on the globe [20]. There are an estimated 0.19–0.88 million km^2^ of global peatlands in the temperate zone (30–50 latitude) [21].

Climate change and other disturbances, such as, landuse change, wetland drainage, coastal flooding, groundwater salinity, and wildfires, alone and in combination, are influencing the C cycle in peatlands [22]. Previously published studies have shown significant ecological changes in peatland C in response to the addition of nitrogen and phosphorus [23, 24], but there has been increasing interest in the role of wildfires in C cycling and altering peatlands from C sinks to C sources. Both nutrient availability and fire regimes are critical regulators of ecosystem structure and function, and modifiers of ecosystem responses to climate change and C storage in peatlands. Whereas nutrient availability is a long-duration and gradual modifier to the C cycle, peatland wildfires are relatively short-duration events and characterized by organic soil combustion and smoldering that occurs over several days to months. A single wildfire may convert peatlands from a C sink to a significant C source, emitting decades to centuries of C sequestered in peatland soils.

In the southeastern United States, there are an estimated 9000 km^2^ of peatlands [25]. Within the southeastern United States, the Coastal Plain of North Carolina is estimated to store 325 terragrams of carbon (TgC) in peat deposits up to 5 m deep [26]. The interannual variability of C emissions from United States temperate peatland fires [26–28], illustrates the limited knowledge of the fragmented spatial pattern of temperate peat deposits which has resulted in a lack of comprehensive data on regional peatland distribution, peat depths, and C sequestration. C emissions from temperate peatland fires are less understood or quantified than large wildfires and their emissions from boreal and tropical peatlands.

The distribution and abundance of plant species that contribute to live and dead fuels, and the development of organic soils within peatland ecosystems is dependent on three ecological factors: soil pH, nutrient availability, and depth to the water table [18, 24, 29, 30]. In the peatlands of North Carolina, USA litter accumulation and decomposition associated with microtopography have resulted in creation of hummocks and depressions across small changes in elevation from sounds, rivers, and streams to inland forests and shrublands. The small elevation changes in microtopography are wildfire ignition areas of origin due to their lower fuel moisture when compared to the surrounding landscape. Peatland microtopography impacts wildfire risk and in combination with the range of soil moisture content determines the species distribution and abundance. However, the ecological factors that contribute to plant community gradients, i.e., recognizable and complex assemblage of plant species which interact with each other as well as with the elements of their environment and is distinct from adjacent assemblages, are complex and vary from site to site. Most temperate peatlands have been disturbed by several anthropogenic activities that confound the controlling influences of microtopography, environmental factors, and wildfires. The uncertainty associated with C loss estimates from wildfires in peatlands across the globe is confounded by the limited knowledge of peatlands, including gradients in nutrient status, natural vegetation communities, areal extent and depth of organic soils, hydrology, and impacts of disturbance.

This study estimates above-ground and below-ground C emissions for vegetation communities from a long-duration temperate peatland wildfire in eastern North Carolina, USA. The objectives of the study were to: (1) estimate above-ground C emissions derived from area burned, fuel loading, pre- and post-burn field vegetation surveys, and fire emission proportions that were characterized for litter, shrub, and tree foliage fractions in United States National Vegetation Classification (USNVC) associations, coupled with tree and shrub density measures, (2) estimate below-ground C emissions utilizing pre- and post-burn Light Detection and Ranging (LIDAR) soil elevation data, soils series classification and C content mapping, remotely sensed burn severity, and pre- and post-burn field soil surveys, and (3) quantify the C emissions from above-ground and below-ground fuel components within individual natural plant communities in a temperate peatland during a long duration wildfire. The study was conducted to improve our understanding of C emissions from above-ground and below-ground fuel components within individual natural plant communities in a temperate peatland during a long-duration wildfire.

## Methods

### Study area

On May 4, 2011 the Pains Bay Fire was ignited by lightning on the United States Fish and Wildlife Service’s (USFWS) Alligator River National Wildlife Refuge, adjacent to Pains Bay on the southern peninsula boundary the Dare County, North Carolina (35.588707°, − 75.803814°). The wildfire ignition occurred in a *Pinus serotina* Michx (pond pine) woodland with no nearby accessible roads. The greatest rate of spread occurred during the first 2 days following ignition and was characterized by a rapidly spreading crown fire in the pond pine woodlands. By June 14, 2011 the wildfire had reached 17,925 ha in size and there were significant areas of organic soil ignition, smoldering, and associated smoke emissions. Organic soil consumption occurred in both the low and high pocosin vegetation throughout the duration of the flaming and smoldering stages of the wildfire. Fire crews conducted fire suppression tactics to extinguish the organic soil fires throughout the smoldering stage of the wildfire with helicopter water drops and hose lay sprinklers supplied by ground pumping stations from nearby water sources. The Pains Bay Fire was declared out after 112 days on August 24, 2011, at which time the wildfire perimeter encompassed 18,329 ha.

The mainland of Dare County, North Carolina, consists of numerous fire-adapted ecosystems. The majority of the land area in mainland Dare County is managed by the USFWS (61,512 ha) as a wildlife refuge and the U.S. Air Force (18,866 ha) as a training area. The Dare County mainland is a peninsula 22.5 km across, bordered on the north by the Albemarle Sound, on the east and south by the brackish Croatan and Pamlico Sounds, respectively, and on the west by the freshwater Alligator River. The long axis of the peninsula extends 46.7 km from north to south.

**Fig. 1:**
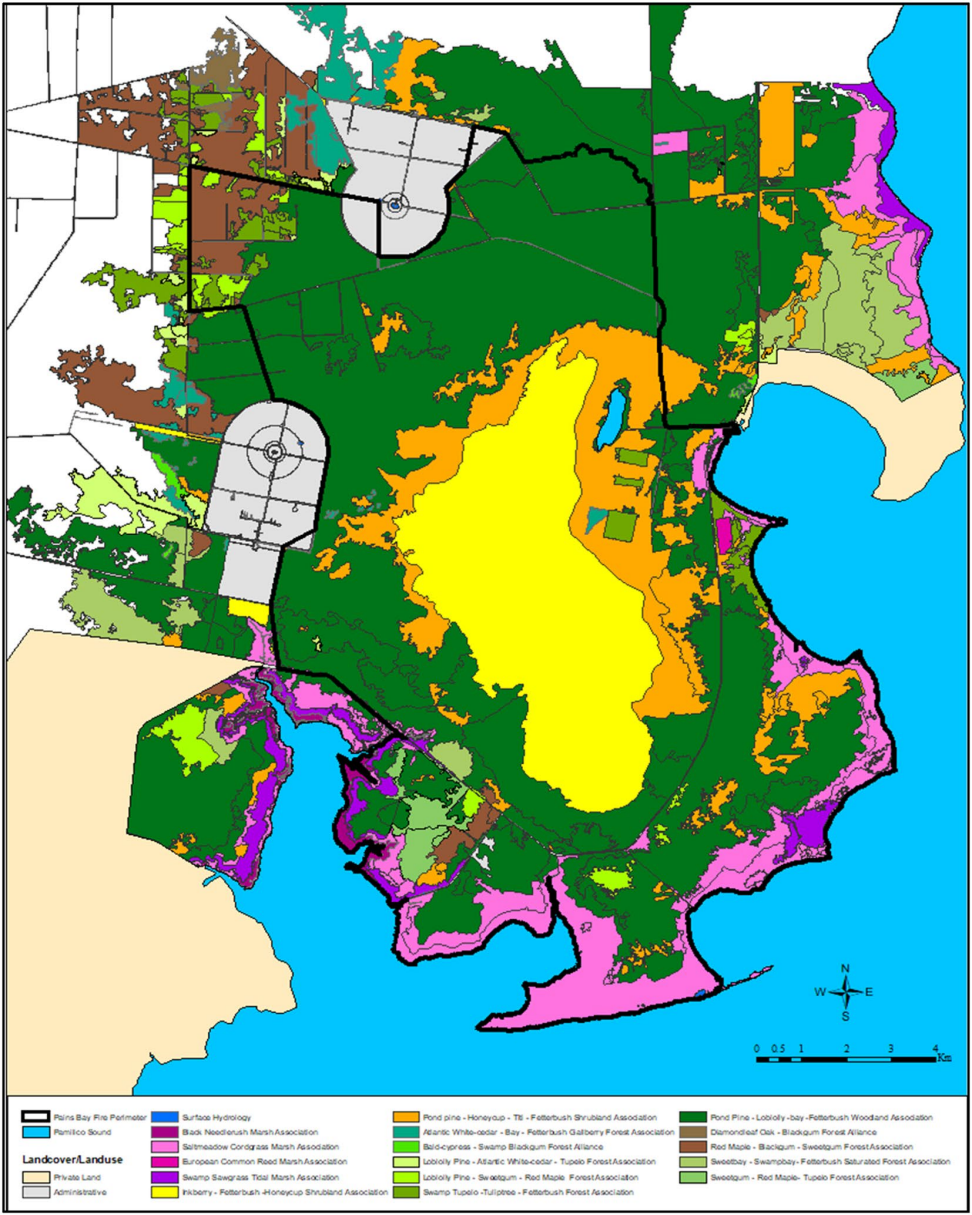
United States National Vegetation Classification within the Pains Bay Fire perimeter (18,329 ha) based on floristics for the Alliance and Association hierarchy for natural vegetation

The climate in the study area is humid subtropical with an average annual temperature of 16.9 °C and an average annual precipitation of 126.9 cm. The vegetation of mainland Dare County has been profoundly affected by wildfire suppression, commercial logging, and sea level rise over the past several centuries [31, 32]. The study area has a pronounced east–west fire frequency gradient based on vegetation influenced by soil elevation above mean sea level, surface hydrology, and groundwater salinity (Fig. 1).

A generalized vegetation gradient from east to west across the peninsula consists of *Spartina patens* (Aiton) Muhl. (saltmeadow cordgrass), *Distichlis spicata* (L.) Greene (saltgrass), and *Juncus roemerianus* Scheele (black needlerush) that form a continuous saltmarsh shoreline band stretching from the Albemarle Sound to the south along the shoreline to the Pamlico Sound. The next vegetation band immediately to the west is comprised of low pocosin vegetation that includes pond pine woodland and an understory of *Arundinaria gigantean* (Walter) Muhl. (giant cane), *Ilex* *glabra* (L.) *A.* *Gray* (little gallberry), and *Lyonia* *lucida* (Lam.) *K.* *Koch* (shining fetterbush). The beginning of salt-intolerant canebrake marks the western limit of storm overwash. The highest elevation central region of the peninsula consists of a low pocosin dome dominated by shrub vegetation [little gallberry, shining fetterbush, and *Zenobia pulverulenta* (*W.* *Bartram* ex Willd.) Pollard (honeycup)] and surrounded by a high pocosin saturated conifer and hardwood forests dominated by *Pinus taeda* L. (loblolly pine), *Nyssa sylvatica* Marshall (blackgum), and *Acer rubrum* L. (red maple). In contrast, the western shore is dominated by non-pyrophytic swamp forests of *Taxodium dichitum* (L.) Rich. (bald cypress), *Chamaecyparis thyoides* (L.) B.S.P. (Atlantic white cedar), and blackgum which fringe the fresh waters of the Alligator River in a narrow band. The high fire frequency saltmarsh and canebreak communities of the eastern side and the low fire frequency river swamp forest on the west comprise the extremes of a cross-peninsula natural fire frequency gradient that ranges from 1–3 years to 100–300 years [33].

### Pre-burn vegetation mapping

A Dare County, North Carolina USA aerial photography mission in spring 2004 collected color-infrared photographs with a spatial resolution of 7.5 inches per pixel [34]. The digitized photographs were orthorectified and used to develop an orthophoto mosaic for use as a base layer during vegetation community mapping. Using the orthophoto mosaic, stereo blockfile, a digital elevation model, surface hydrology data, and a digital soil survey, polygons representing distinct vegetation communities were delineated into fourteen association level communities of the USNVC within the fire perimeter (Fig. 1). The USNVC is an ecosystem-based classification scheme in which vegetation communities are grouped by their characteristic physiognomy and floristic composition [35]. To differentiate vegetation types on the orthophoto mosaic and stereo analyst block files, seven photogrammetric interpretation attributes were used: size, shape, shadow, color, texture, pattern, and association with other objects [36]. The heads-up stereo photography allowed differentiation of vegetation communities with differing dominant tree heights, canopy shapes, and canopy closure, the critical strata used to discriminate between USNVC Associations [37]. Soil series, above mean sea level elevation, and hydrologic soil groups were used to further inform the vegetation classification.

The fourteen associations were grouped into four forest, woodland, shrub, and herb vegetation classes: pine/hardwood swamp forest, pine woodland, shrubland, and saltmarsh. The four vegetation classes comprise the highest to lowest elevation gradient and the dry to wet surface hydrology regimes within the study area (Table 1).

**Table. 1:** Burned area (ha) of major vegetation classes and their corresponding dNBR burn severity classes

Vegetation classes	dNBR fire severity classes (ha)				Total burned area (ha)
	Unburned/very low severity	Low severity	Moderate severity	High severity	
Shrubland	13.7	44.4	1074.4	4549.7	5682.2
Pine woodland	219.0	1144.1	3012.2	4697.1	9072.4
Pine/hardwood swamp forest	465.7	198.1	339.3	182.7	1185.8
Salt marsh	822.7	433.4	659.1	129.9	2045.1

The pine/hardwood swamp forests were associated with Hyde series loam and Roper series muck soils, consisting of poorly drained soils formed over loamy marine sediments. Woodlands were found on Belhaven and Ponzer series muck soils, poorly drained soils that formed over loamy marine sediments. The shrubland vegetation was located on Pungo series muck soil, poorly drained soils that formed in organic material over loamy or clayey marine sediments. The saltmarsh vegetation was associated with the Currituck series mucky peat soil, a frequently tidally flooded poorly drained soil that formed in organic material over sandy marine sediments.

### Differential Normalized Burn Ratio (dNBR) burn severity damage classes

Fire damage categories were defined based upon burn severity and USNVC Association vegetation classifications. Burn severity categories were selected ranging from 0 (no damage) to 3 (most severe damage). dNBR values [38] were classified into four BARC-A fire damage categories using the Jenks Natural Breaks Classification method [39, 40]. For our study site, the four burn severity damage class thresholds were: unburned/very low severity  =  0–134, low severity  =  135–168, moderate severity  =  169–246, and high severity  =  247–255. The mosaic image ArcGIS grid cells were intersected with land use/land cover polygons to illustrate burn severity within each of fourteen USNVC associations. ArcGIS^®^ 10.1 [41] was used to carry out spatial processing for burn severity damage classes in a UTM 18 North projection with the WGS84 datum. Additional tabular processing was done using SAS^®^ 9.2 [42].

**Fig. 2:**
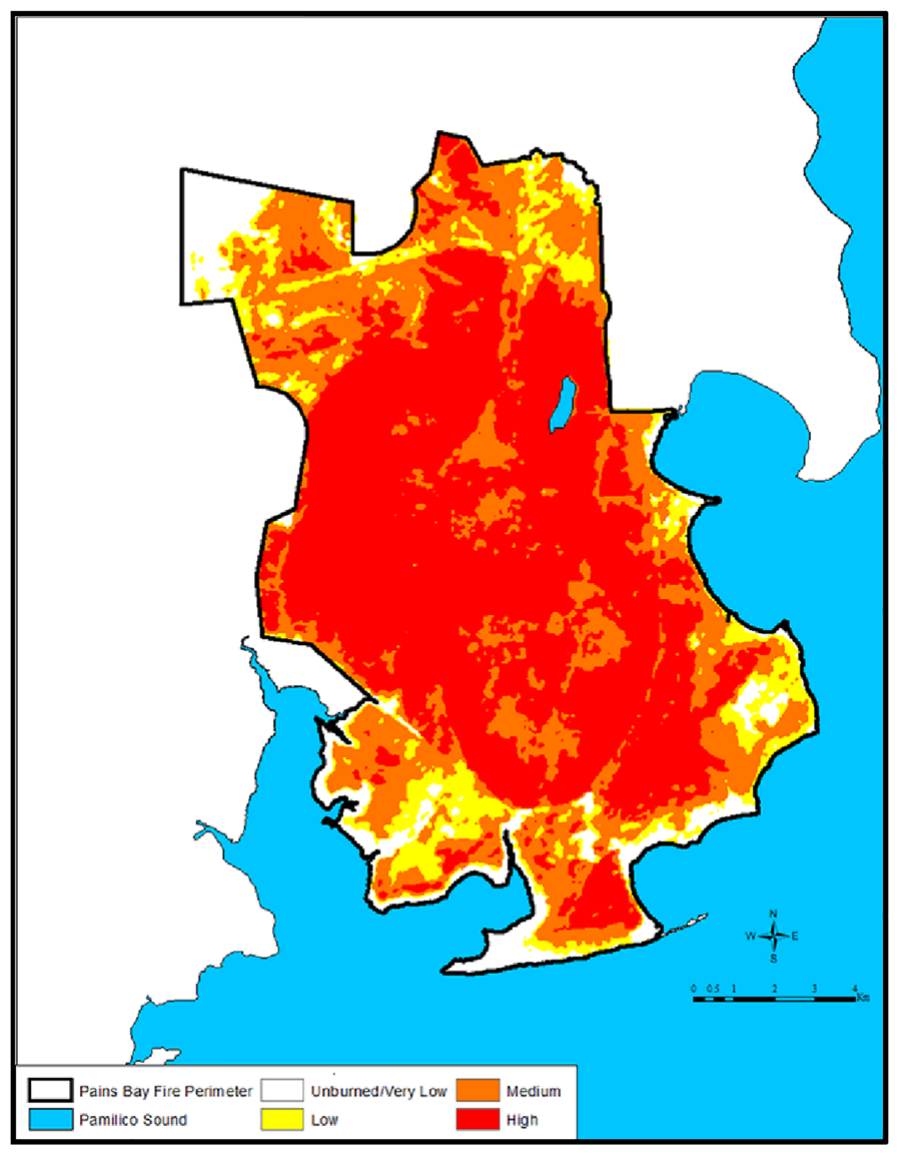
Differential Normalized Burn Ratio (dNBR) mosaic image product within the perimeter of the Pains Bay Fire illustrating the Burned Area Reflectance Classification thresholds: unburned/very low severity  =  0–134, low severity  =  135–168, moderate severity  =  169–246, and high severity  =  247–255

The normalized burn ratio or Burned Area Reflectance Classification (BARC) grids based on Landsat images from 5 dates were supplied by USGS, along with grids containing the processed burn ratios. The pre-fire image was taken on May 8, 2010, and the normalized burn ratios were calculated relative to this pre-fire image. Images taken during the 2011 Pains Bay Fire on May 20th, July 4th, 20th, and 28th were used to calculate the normalized burn ratios. The initial burn ratio grids were scaled from 0 to 255. Since the wildfire was of long duration, some areas were exhibiting herbaceous vegetation regrowth while other areas within the fire perimeter were in flaming and smoldering stages. We used burn ratio grids from all four dates to calculate a new combined image product of maximum burn ratio within the fire perimeter from May 20th to July 28th (Fig. 2). The maximum value was identified for each collocated grid cell in the four image dates. The grids, coded from 0 to 255, were smoothed with a 3  ×  3 median filter. The grids were then reclassified to the four burn severity damage classes and again smoothed with a 3  ×  3 median filter. Visual inspection indicated that other than smoothing there were no distortions of the original values. The final combined image product combined maximum burn severity for each cell within the entire Pains Bay Fire perimeter.

### Organic soil ignition detection

**Fig. 3:**
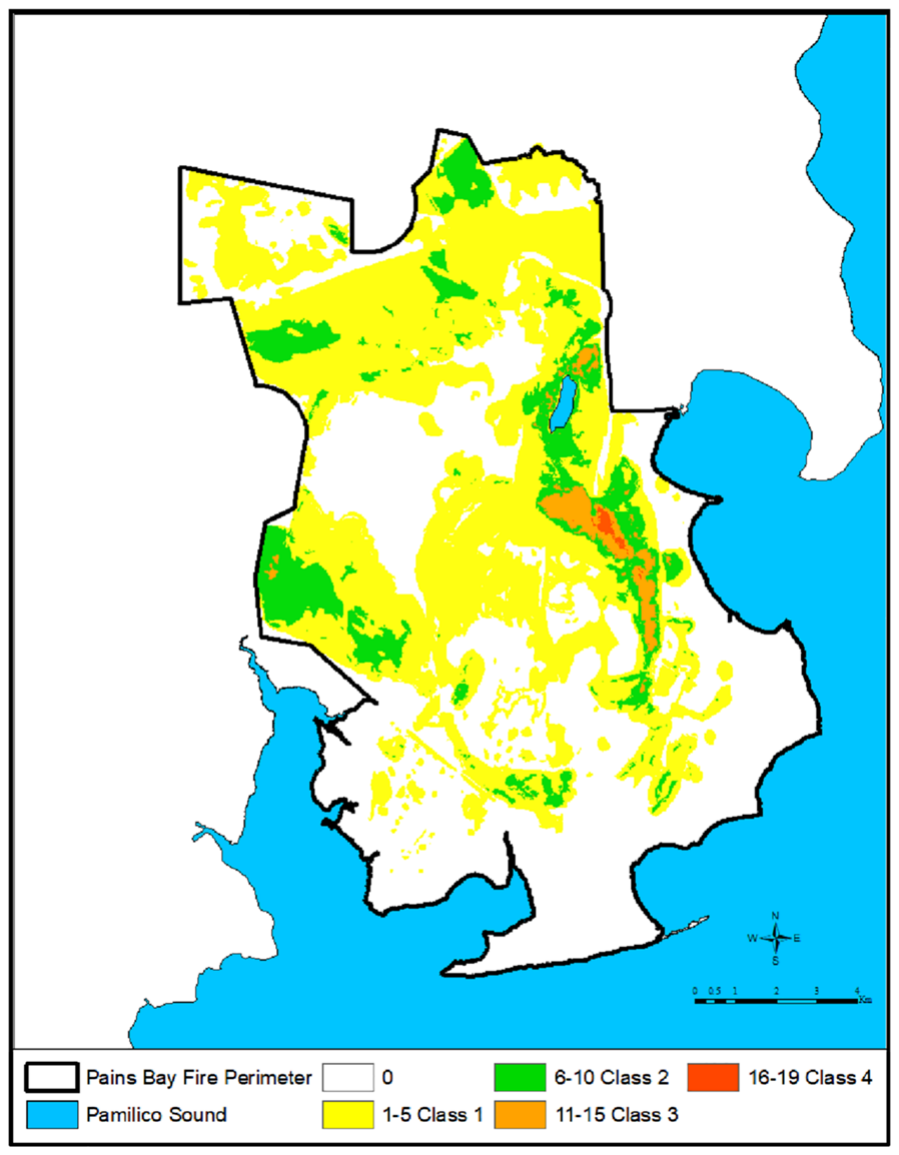
Organic soil ignition detection mosaic image derived from USFWS and NCDFR aerial detections, and USFS Firehawk data accessed from the NIROPS, illustrating ground fire detection class ranges: 0, 1–5, 6–10, 11–15, and 16–19

The measurement of soil organic-matter emission losses during and following wildfires assists in quantifying changes in C cycling. Organic soil ignitions were determined using aerial thermal imagery from the US Fish and Wildlife Service (USFWS) and North Carolina Division of Forest Resources (NCDFR), and Firehawk infrared data accessed from the US Department of Agriculture Forest Service’s National Infrared Operations (NIROPS). The NCDFR provided vector layers representing the occurrence of intense ground fires and scattered fires in the study area for 21 dates during the fire. Most dates had separate data representing these two classes. The intense class indicates a visible solid block of burning fuel. The scattered fire class represents an area where there were pockets of ground fire and possibly some active surface fire. The early imagery (5 dates) was collected by the NCDFR via helicopter. The later 15 dates were based on thermal imagery accessed from NIROPS. Data from one date was not used because the scattered and intense classes were not available separately. The fire layers were rasterized individually using a 5  ×  5 m cell size aligned with the normalized burn grids. The grids were assigned a 1 where there was intense or scattered fire on that date, and a 0 where no fire was identified. The grids were overlaid and summed for each fire-detect location. For the 20 dates with ground fire detects, there were up to 19 days where ground fire was identified in an adjoining cluster of grid cells. Each cell was classified into one of five classes based on the number of days with infrared detected ground fire ignition. All fire detect grid cells were merged into one ESRI ArcGIS layer and polygon overlaps were analyzed into five classes: class 0  =  no detects, class 1  =  1–5 detects, class 2  =  6–10 detects, class 3  =  11–15 detects, and class 4  =  16–19 detects. A mosaic image of the fire detects (Fig. 3) was developed to illustrate the duration of ground fire and to remove the effects of vegetation greening following the active flaming and smoldering stages of the wildfire over the duration of the 21 observation dates (Fig. 3).

### Pre- and post-burn bare earth elevation measurements

Pre- and post-fire elevation difference measurements were used to determine organic soil loss. Pre-fire elevation data were derived using bare earth points from the North Carolina Flood Mapping Program [43]. LIDAR returns were acquired for January to March of 2001. Vertical accuracy was  <  20 cm Root Mean Square Error (RMSE). A random point was selected on the ARNWR for each vegetation class and each burn severity damage class within the vegetation class that was greater than or equal to 100 acres. East to west transects were drawn in ArcGIS and a rectangular polygon was drawn along each transect to include a minimum of 50 LIDAR ground points. Post-fire soil elevation was measured at 50 randomly-selected bare earth points along each of the east–west transects using survey-grade Global Positioning System (GPS). The system employed a Trimble R4 GPS Receiver—A Base Station and Rover Receiver for RTK GPS/GNSS Surveying, a Trimble TSC2 Controller and Trimble Survey Controller Software, and a Trimble RTX Verizon Cellular Data Correction Services – Cellular Network of GNSS Reference. Field measurements were corrected to the National Spatial Reference System using the NOAA Online Position User Service (OPUS). Similar approaches to assess LIDAR elevation accuracy have been conducted in other contexts [44, 45] and in the soil and vegetation assessments for the Evans Road Fire [27]. Photo images were collected for each USNVC association and each damage class to document the post-fire vegetation and soil damage.

### Estimation of below-ground organics soil C emissions

Below-ground soil C emissions were calculated from GIS-derived area of land cover category combinations for the North Carolina Dare County and the SSURGO soil database elements (soil series, organic soil horizon depth, bulk density, and C content) [46]. Mean depth of organic soil horizon depth changes were calculated based on the difference of pre-burn LIDAR data points and post-burn field survey of co-located points.

Soil series within the fire perimeter consisted of seven organic soil series [46] with a thickness of the organic layers that ranged from 129.5 cm to 203.2 cm: Belhaven (Loamy, mixed, dysic, thermic Terric Haplosaprists); Currituck (Sandy or sandy-skeletal, mixed, euic, thermic Terric Medisaprists), Hobonny (Euic, thermic Medisaprists), Hyde (Fine-silty, mixed, thermic Umbraquults), Ponzer (Loamy, mixed, dysic, thermic Terric Haplosaprists), Pungo (Dysic, thermic Typic Haplosaprists); and Roper (Fine-silty, mixed, semiactive, acid, thermic Histic Humaquepts)).

The Microsoft Office Access database was used to import the map unit, soil physical and chemical components, and soil component horizon tables. These tables are related in the following manner: each map unit has one or more components, and each component has one or more horizons. We calculated averages for representative bulk density and representative percent organic matter within components from the data for the uppermost horizon within each component. The average values for each component were used to calculate weighted averages for each map unit using the component percentage value. The map unit averages were manipulated in ArcGIS 10.0 [41] and joined to a GIS shapefile showing the extents of each soil map unit, using the common map unit key column in each dataset. Soils GIS data were downloaded from the Soil Data Mart. The soil data were clipped to the fire boundary, and intersected with the damage and ownership classes. After calculating acreage for the clipped and intersected polygons, soil organic C was scaled up to the polygon area.

Soil organic C was calculated as adapted from Rasmussen [47] and Tan et al. [48] as follows:

1 $${\text{SOCi = }}\left[ {{\text{Di}} \times \rho {\text{bi}} \times \left( {{\text{OMi}} \times 0.5} \right)/100} \right] \times 10$$

where SOCi is the soil organic C content (kg/m^2^) for the O or Oa horizon; Di is the depth of soil consumed (cm); ρbi is the soil bulk density (g/cm^3^); and OMi is the organic matter weight percentage. Soil C emissions (tons C) were estimated for each of the damage classes delineated for the Pains Bay Fire. Within each damage class, C emissions were summed for each of the NVCS Association, soil series, area, and mean depth of consumption.

### Estimation of pre- and post-burn above-ground biomass

The estimation of C emissions from wildfires was first described by Seiler and Crutzen [49] and modified by French et al. [50]. We modified the multistep process to include: (1) determination of the area burned by relating the fire perimeter to the USNVC vegetation class and land cover class; (2) geospatial linkage of C stocks associated with land cover classifications to the C stocks in soil series and their horizons; (3) estimation of above- and below-ground C consumption and emissions the C fractions for 14 USNVC vegetation associations; and (4) estimation of C emissions from 7 soil series. Consumed C fractions were estimated for USNVC association and soil series. The estimation of the total C release (Ct) from burning of both above-ground biomass and ground layers was based on the equation modified by French et al. [51] from Seiler and Crutzen [49]:

2 $${\text{Ct}} = {\text{A}}\left( {{\text{Ca}}\beta {\text{a}} + {\text{Cg}}\beta {\text{g}}} \right)$$

where A is the total area burned (ha); Ca is the average C content of above-ground biomass (kg C m^−2^) assuming the C fraction of the above-ground biomass is about 0.50; βa is the fraction of above-ground biomass consumed during a fire; Cg is the C content (kg C m^−2^) of soil horizons exposed to a fire, and βg is the fraction of the soil horizon consumed by the fire.

Fire intensity on the Pains Bay Fire varied across space, resulting in heterogeneous fuel consumption across the USNVS association vegetation types. Estimates of the above-ground C consumption were conducted within each vegetation Association using differences for each of the four dNBR values. Higher dNBR values indicate higher above-ground combustion fraction. Litter, shrub, and tree foliage combustion fractions were determined for each dNBR value within each of the USNVC associations.

Above-ground C emissions were estimated using area burned, fuel loading (biomass per unit area), and consumption proportions following various studies addressing biomass combustion [49, 51, 52]. Biomass calculations were then multiplied by 0.5 to attain estimates of C. Land cover classification [34] estimates informed (1) the area burned, and (2) the basis of the fuel loading figures. Above-ground C emissions were calculated from estimates of tree density, and tree foliage, litter, and shrub biomass.

Foliage biomass estimates employed allometric biomass equations multiplied by a foliage ratio equation for eastern conifers and red maple: the dominant evergreen and deciduous species in the study area. The eastern conifer biomass equation per tree was:

3 $${\text{Tree biomass }} = \left\{ {0.5 + \left[ {15000^{ * } {\text{d}}2.7/} \right.\left. {\left( {{\text{d}}2.7 + \left. {364,946} \right)} \right.} \right]} \right\}$$

where d was the average stand diameter [53]. Equation results were multiplied by a foliage ratio using an equation for softwoods:

4 $${\text{Foliage ratio}} = \exp \left[ { - 2.9584 + \left( {4.4766/{\text{d}}} \right)} \right]$$

where d was the average stand diameter [54]. Red maple biomass was derived from the following equation for individual trees:

5 $${\text{log1}}0{\text{ tree biomass}} = \left\{ {{ - }0.{86}0{2} + {1}.{7963}^{*} \left[ {{\text{log1}}0\left( {\text{d}} \right)} \right]} \right\}$$

where d was the average stand diameter [55]. Equation results were multiplied by a foliage ratio using an equation for hardwoods:

6 $${\text{Foliage ratio}} = {\text{exp}}\left[ {{ - }{4}.0{813} + \left( {{5}.{8816}/{\text{d}}} \right)} \right]$$

where d was the average stand diameter [54]. Average stand diameters and tree densities were measured within each of the forest and woodland vegetation associations using variable radius plots at each sample point and applied to equations in order to obtain biomass on a per area basis. Land area was applied to equations in order to obtain biomass on a per area basis [56].

## Results

### Pains Bay Fire: fire behavior and vegetation associations

The largest total area burned, and moderate and high fire severity classes were in the shrubland and pine woodland vegetation classes. These vegetation classes reflect a natural plant gradient governed by elevation and surface hydrology. The shrubland class was found on the highest elevation and the lowest depth to the water table on the Dare County peninsula. The shrubland comprise the low pocosin dome which under extreme burning conditions fires will burn into the organic layer and typically burn all above-ground vegetation. Most shrubs will resprout vigorously from roots and rhizomes and regain 20% of their pre-fire biomass within one growing season. Some plants, such as *Zenobia* and various herbs, recover quickly and are dominant for several years until other shrub species reestablish themselves. Pine woodlands are dominated by pond pine, a classic example of a fire adapted species with the ability to sprout from either the root crown or epicormic buds along the bole and branches, and having serotinous cones that release seeds post-fire. When wildfires occur during periods of low surface water, mortality is high in the tree, shrub, and herbaceous vegetation and organic soil combustion is common. Fires change the relative species composition, favoring species that recover quickly. However, intense heat during the flaming stage of the Pains Bay Fire resulted in the development of a hydrophobic crust on the surface of the peatland soils that delayed vegetation regrowth. Species diversity was highest post-fire and gradually declined thereafter.

The largest high severity class area (4697.1 ha) and total burned area (9072.3 ha) was the pine woodland vegetation class which was comprised of one USNVC association, the Pond Pine—Loblolly-bay/Shining Fetterbush Swamp Woodland Association. The Pains Bay Fire was ignited by a lightning strike in the pine woodland and burned northward consuming 239 ha on day one of the fire. On day two of the fire, the flaming stage burned to the south into the salt marsh until reaching the shoreline of the Pamlico Sound (882 ha). The wind direction returned to the north on day three of the fire and the fire spotted across a county highway. The flaming stage exhibited extreme fire behavior and a running crown fire in the pond pine woodland that consumed 2555 ha. The extreme fire behavior on day three resulted in the combustion of all above-ground vegetation and the ignition of the peatland soils. The resulting flaming and groundfires moldering ignited the pine woodland and saltmarsh to the east until reaching the shoreline of the Pamlico Sound and to the west into the shrubland vegetation, that consumed 4982 ha on day four of the fire. The active flaming wildfire stage of the fire consumed 47% of the total area burned during the first four days (May 5–8, 2011).

The remaining active flaming stages of the fire (May 9–June 13, 2011) were comprised of a number of planned back firing and burnouts using existing county highway, gravel roads, and the peninsula shoreline as fire lines to contain the managed firing operations. Many of the burnout operations resulted in crown fires adjacent to roads in moderate and high fire severity dNBR values in the shrubland and pine woodland classes. The backfiring and burnouts were conducted to contain potential wildfire spread into the wildland urban interface (WUI) and prevent destruction of private properties in fishing communities located to the east, north, and south of the Pains Bay Fire, and to infrastructure on the western boundary of the fire. The organic soil groundfires continued until the Pains Bay Fire was declared out after 112 days on August 24, 2011.

### USNVC vegetation association communities and fire severity

The largest woodland burn severity vegetation association was the Pond Pine—Loblolly-bay/Shining Fetterbush Swamp Woodland Association (9072.2 ha). Combined with the adjacent shrubland communities with the highest burn severities, the Pond pine/Honeycup—Swamp Titi -Shining Fetterbush Wooded Wet Shrubland Association (3265.5 ha) and the Inkberry—Shinning Fetterbush—Honeycup Wet Shrubland Association (2396.3 ha), the three vegetation communities comprised 80.5% of the total land area within the fire perimeter (Table 2).

**Table 2:** Area (ha) of land cover/land use, The US National Vegetation Classification Associations, differenced Normalized Burn Ratio (dNBR) classes, and ground fire frequency classes

Land cover/land use and The US National Vegetation Classification Associations^a^	Differenced Normalized Burn Ratio classes																			
	Unburned/very low severity (0)					Low severity (1)					Moderate severity (2)					High severity (3)				
	Ground fire frequency classes^b^																			
	0	1	2	3	4	0	1	2	3	4	0	1	2	3	4	0	1	2	3	4
Surface waters	13.8	0.8	0	0	0	40.5	3.6	0	0	0	17.8	10.1	0	0	0	0.8	1.2	0	0	0
Developed lands	36.0	4.9	3.2	0	0	61.5	17.8	4.4	0	0	69.6	29.5	3.6	0	0	19.8	6.1	0	0	0
Atlantic White-cedar/Swamp Bay/Shining Fetterbush—Large Gallberry Swamp Forest Association	0.0	0.0	0	0	0	0	0	0.4	0	0	0	0	5.7	0.4	0	5.2	2	3.6	4.8	0
Black Needlerush Salt Marsh Association	81.3	0.0	0	0	0	0.8	0	0	0	0	0	0	0	0	0	0	0	0	0	0
European Common Reed Eastern Ruderal Marsh Association	2.8	0.0	0	0	0	8.1	0	0	0	0	14.9	0	0	0	0	0	0	0	0	0
Loblolly Pine—Atlantic White-cedar—Swamp Tupelo/Shining fetterbush—Coastal Sweet-pepperbush Swamp Forest Association	0.0	0.0	0	0	0	1.6	0	0	0	0	0	0	0	0	0	0	0	3.6	0	0
Swamp Tupelo—Tuliptree—(Pond Pine, Loblolly Pine)/Shining Fetterbush Swamp Forest Association	50.9	40.8	0	0	0	57.4	20.2	0	0	0	48.5	38	2	0	0	1.2	23.8	18.6	39.2	0
Loblolly Pine—Red Maple—Sweetgum/Switch Cane Ruderal Wet Forest Association	42.9	31.6	0	0	0	10.9	3.6	0	0	0	40.8	10.9	0	0	0	19	0.8	0	0	0
Pond Pine—Loblolly-bay/Shining Fetterbush Swamp Woodland Association	177.7	40.9	0.4	0	0	610.7	528.5	4.9	0	0	1191.3	1575.8	239.9	5.2	0	1286.1	2436.2	873.3	85.7	15.7
Saltmeadow Cordgrass—Saltgrass—(Black Needlerush) Salt Marsh Association	644.7	0.8	0	0	0	382	1.2	0	0	0	562.5	7.2	0	0	0	126.3	2	1.6	0	0
Swamp Sawgrass Tidal Salt Marsh Association	93.1	0	0	0	0	41.3	0	0	0	0	74.5	0	0	0	0	0	0	0	0	0
Inkberry—Shining Fetterbush—Honeycup Wet Shrubland Association	0.0	0.0	0	0	0	0	0	0	0	0	422.8	280.4	0	0	0	1182.9	1363.7	15.7	0	0
Pond pine/Honeycup—Swamp Titi—Shining Fetterbush Wooded Wet Shrubland Association	12.9	0.8	0	0	0	40.5	3.6	0	0	0	228.2	97.9	21.4	3.6	0	612.7	848.6	372.3	143.3	10.5
Red Maple—Blackgum species—Sweetgum—Water Oak/Royal Fern species Swamp Forest Association	184.5	110.1	0	0	0	44.5	23.9	0	0	0	14.6	9.3	0	0	0	2.4	0	0	0	0
Sweetbay—Swampbay Swamp/Shining Fetterbush Swamp Forest Association	0.4	0	0	0	0	1.2	0	0	0	0	9.3	4.8	0	0	0	24.7	13.7	0	0	0
Sweetgum—Red Maple—Swamp Tupelo/Cypress Swamp Sedge Swamp Forest Association	4.1	0.4	0	0	0	23.1	11.3	0	0	0	108.1	46.9	0	0	0	12.5	7.6	0	0	0
Total area	1345.0	231.0	3.64	0	0	1324.1	613.7	9.7	0	0	2802.9	2110.8	272.6	9.2	0	3293.6	4705.7	1288.7	273	26.2

^a^CEGL006146 Atlantic White-cedar/Swamp Bay/Shining Fetterbush—Large Gallberry Swamp Forest Association (*Chamaecyparis thyoides*/*Persea palustris*/*Lyonia lucida*—*Ilex coriacea *Swamp Forest Swamp Forest Association), CEGL004186 Black Needlerush Salt Marsh Association (*Juncus roemerianus* Salt Marsh Association), CEGL004141 European Common Reed Eastern Ruderal Marsh Association (*Phragmites australis *ssp. Australis Eastern Ruderal Marsh Association), CEGL004458 Pond pine/Honeycup—Swamp Titi—Shining Fetterbush Wooded Wet Shrubland Association (Pinus serotina/*Zenobia pulverulenta—Cyrilla racemiflora—Lyonia lucida* Wooded Wet Shrubland Association), CEGL007558 Loblolly Pine—Atlantic White-cedar—Swamp Tupelo/Shining fetterbush—Coastal Sweet-pepperbush Swamp Forest Association (*Pinus taeda*—*Chamaecyparis thoides*—*Nyssa biflora/Lyonia lucida*—*Clethra alnifolia* Swamp Forest Association), CEGL004734 Swamp Tupelo—Tuliptree—(Pond Pine, Loblolly Pine)/Shining Fetterbush Swamp Forest Association (*Nyssa biflora*—*Liriodendron tulipifera*—*Pinus (serotina*, *taeda)*/*Lyonia lucida *Swamp Forest Association), CEGL004649 Loblolly Pine—Red Maple—Sweetgum/Switch Cane Ruderal Wet Forest Association (*Pinus taeda*—*Acer rubrum*—*Liquidambar styraciflua/Arundinaria tecta* Ruderal Wet Forest Association), CEGL003671 Pond Pine—Loblolly-bay/Shining Fetterbush Swamp Woodland (*Pinus serotina*—*Gordonia lasianthus/Lyonia lucida* Swamp Woodland), CEGL004197 Saltmeadow Cordgrass—Saltgrass—(Black Needlerush) Salt Marsh Association (*Spartina patens*—*Distichlis spicata* – (*Juncus roemerianus*) Salt Marsh Association), CEGL004178 Swamp Sawgrass Tidal Salt Marsh (*Cladium mariscus* Tidal Salt Marsh), CEGL003944 Inkberry—Shining Fetterbush—Honeycup Wet Shrubland Association (*Ilex glabra*—*Lyonia lucida* – *Zenobia pulverulenta* Wet Shrubland Association), CEGL007982 Red Maple—Blackgum species—Sweetgum—Water Oak/Royal Fern species Swamp Forest Association (*Acer rubrum*—*Nyssa* spp.—*Liquidambar styraciflua*—*Quercus nigra/Osmunda *spp. Swamp Forest Association), CEGL007049 Sweetbay—Swampbay Swamp/Shining Fetterbush Swamp Forest Association (*Magnolia* *virginiana*—*Persea palustris/Lyonia lucida* Swamp Forest Association), CEGL006223 Sweetgum—Red Maple/Wax-myrtle Mid-Atlantic Swamp Forest Association (*Liquidambar styraciflua—Acer rubrum*—*Nyssa biflora/Carex joorii* Swamp Forest Association)

^b^The five fire frequency classes are: class 0  =  no detects, class 1  =  1–5 detects, class 2  =  6–10 detects, class 3  =  11–15 detects, and class 4  =  16–19 detect

**Fig. 4:**
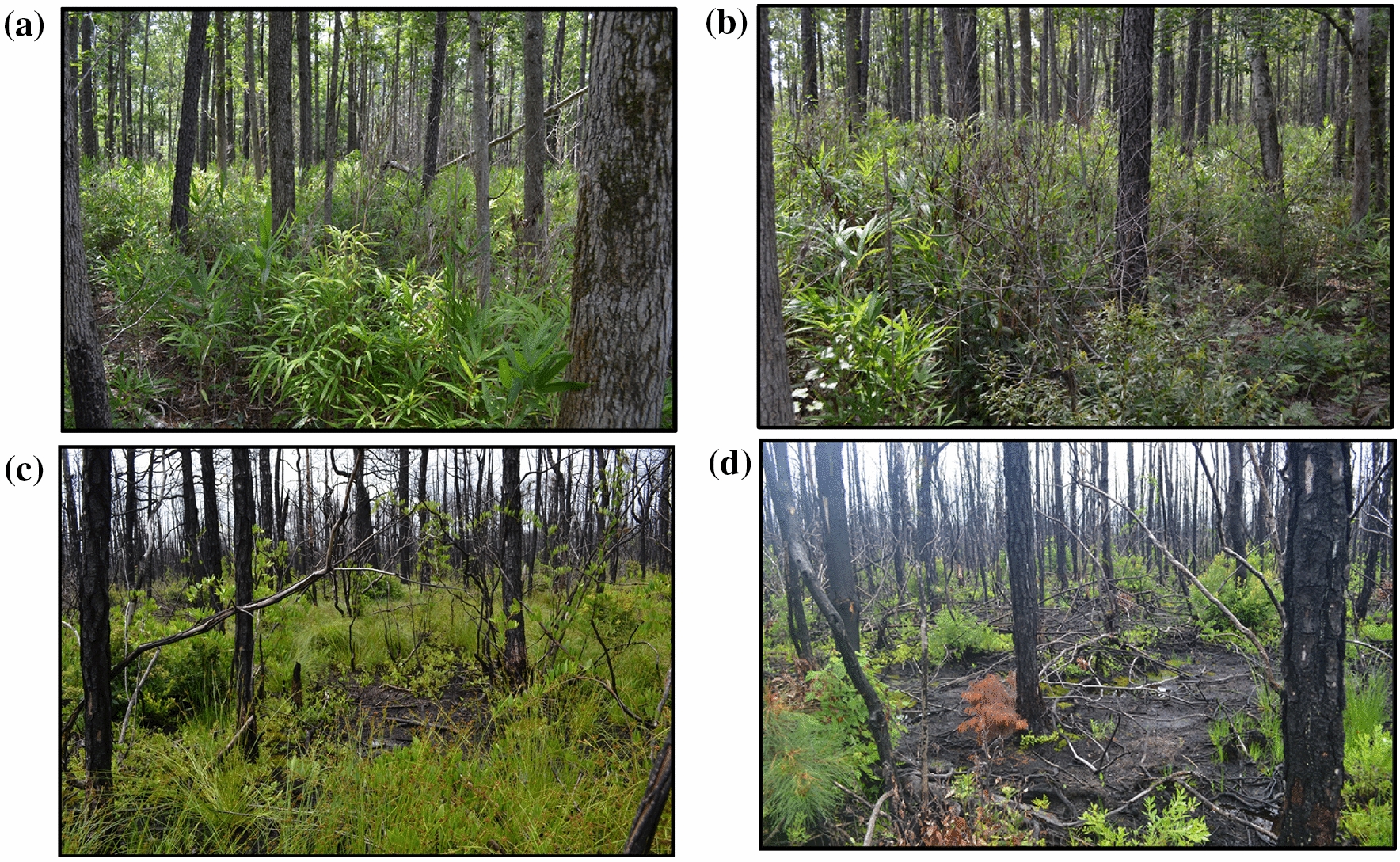
Pond pine—Loblolly-bay/Shining Fetterbush Swamp Woodland Association differenced normalized burn ratio (dNBR) classes: **a** Class 0, **b** Class 1, **c** Class 2, and **d** Class 3. (Note: Fig. 4d illustrates smoke emission from small pockets of smoldering stage organic soil combustion 4 months following the fire ignition and the onset of vegetation regreening)

The burn severity damage and subsequent regreening of vegetation and the seedbed in the four BARC-A groups of the Pond Pine—Loblolly-bay/Shining Fetterbush Swamp Woodland Association, four months after the fire ignition fire are shown in Fig. 4. The pond pines in burn severity Class 2 (Fig. 4d) and Class 3 (Fig. 4c) had 100% tree, shrub, and herbaceous top kill. Although pond pine is a fire adapted tree species with serotinous cones, the running crown fire combusted all of the tree foliage, cones, and small branches. The flaming stage heating killed the crown branches and the exposed tree boles. There was limited epicormic shooting at the root collar and organic soil interface, and limited sprouting of shrubs from below-ground vegetative structures following the suppression of ground fires. In the burn severity Class 3, the regreening was more extensive and occurred from below-ground vegetative structures and revegetation from seeds in patches of unburned organic soil. Burn severity Class 1 (Fig. 4b) was characterized by patchy litter combustion and associated shrub and herbaceous mortality in areas with larger quantities of litter and fine woody debris. Burn severity Class 0 (Fig. 4a) had no shrub or tree mortality and the wildfire effects were confined to small areas of patchy litter combustion that were ignited from flaming stage airborne fire brands.

Six vegetation communities were classed into salt marsh and swamp forest. The salt marsh vegetation communities had daily tidal standing water. The salt marsh vegetation communities were spatially distributed along a slight elevation gradient which began at the open water and land interface with the Black Needlerush Salt Marsh Association and transitioned into the Saltmeadow Cordgrass – Saltgrass – (Black Needlerush) Salt Marsh Association and the Swamp Sawgrass Tidal Salt Marsh Association. The European Common Reed Eastern Ruderal Marsh Association was found at the highest elevation of the salt marsh and predominantly on the banks of tidal influenced streams at the salt marsh to woodland transition zone. The salt marsh vegetation associations contributed to the development of the Currituck mucky peat and the Belhaven muck soil series.

Swamp forests occur in areas of seasonal or intermittent standing water. The standing water and high litter and woody debris surface fuel moistures contributed to the predominant unburned and low fire severity classifications for these vegetation communities. The Red Maple—Blackgum species—Sweetgum—Water Oak/Royal Fern species Swamp Forest Association, Sweetbay—Swampbay Swamp/Shining Fetterbush Swamp Forest Association, Sweetgum—Red Maple—Swamp Tupelo/Cypress Swamp Sedge Swamp Forest Association, and the Swamp Tupelo—Tuliptree—(Pond Pine, Loblolly Pine)/Shining Fetterbush Swamp Forest Association located in isolated depressions in woodlands and on the western perimeter of the Pains Bay Fire.

Swamp forest vegetation communities were associated with Currituck muck peat, Roper muck, and Ponzer muck soil series which are characterized by an organic surface layer with underlying loam and sand. Swamp forests were 6% of the total area of vegetation communities and 49% of area were in the unburned/low severity dNBR fire severity class. The moderate and high severity classes were located on vegetation community transition boundaries swamp forest and woodlands with extreme fire behavior.

### Organic soil ignition and dNBR

The combustion and smoldering of peatland soils determined the revegetation of wetland species within their natural range of soil moisture content, as determined by the depth to the water table, soil pH, and nutrient availability. The distribution and abundance of plant species that contribute to live and dead fuels, and the development of organic soils by peatland species within their natural range of soil moisture content, are determined by the depth to the water table, soil pH, and nutrient availability. The distribution and abundance of plant species that contribute to live and dead fuels, and the development of organic soils within peatland ecosystems is influenced by wildfires and the soil moisture status at the time of ignition. Wildfires that ignite when soil moisture content is high result in above-ground combustion of dead and live woody and herbaceous vegetation, and litter and fine woody debris. In contrast, wildfires that ignite during periods of low soil moisture content typically result in organic soil combustion and long-duration smoldering. These fires result in the mortality of trees and shrubs by the flaming stage ignition of boles and branches, and the smoldering stage conduction and radiation heating of above- and below-ground vegetative structures.

Analyses of the remotely sensed thermal infrared energy for the Pains Bay Fire found that the higher ground fire frequency classes were associated with the moderate and severe dNBR classes (Fig. 3), however the spatial extent of the moderate and severe dNBR did not correlate with the limited spatial extent of the higher ground fire frequency classes.

### Carbon emissions

The Pains Bay Fire total above-ground C emissions were 2,89,579 t C for the fourteen USNVC vegetation association communities and 214 t C ha^−1^ for the burn area. Above-ground emissions were comprised of litter (69,656 t C), shrub (1,68,983 t C), and foliage (50,940 t C) (Table 3). The largest emissions were in the pine woodland Pond Pine—Loblolly-bay/Shining Fetterbush Swamp Woodland Association (1,35,745 t C) and in its highest fire severe class (82,898 t C). The next highest C emissions were in the adjacent shrubland Pond pine/Honeycup—Swamp Titi—Shining Fetterbush Wooded Wet Shrubland Association (83,355 t C) and the Inkberry—Shining Fetterbush—Honeycup Wet Shrubland Association (60,354 t C). These three USNVC vegetation association comprised 96.5% of the total above-ground C emissions and 80.5% of the total area within the fire perimeter for the Pain Bay Fire. The salt marsh association had combined C emissions of 2.4% of the total above-ground C emissions and 11.2% of the total land area within the fire perimeter. The remaining pine/hardwood swamp forests had 1.1% of the total above-ground C emissions and 8.3% of the land area.

**Table 3:** Above-ground C emissions (t C) by National Vegetation Classification Associations and differenced Normalized Burn Ratio (dNBR) classes

National Vegetation Classification Association^a^	dNBR classes^b^	Area (ha)	Litter (t C)	Shrub (t C)	Foliage (t C)	C emissions (t C)	C emissions (t C ha^−1^)
Black Needlerush Salt Marsh Association	0	81.3	0	68	0	68	0.84
	1	0.8	0	0	0	0	0.00
	2	0.0	0	0	0	0	0.00
	3	0.0	0	0	0	0	0.00
Pond pine/Honeycup—Swamp Titi—Shining Fetterbush Wooded Wet Shrubland Association	0	0.0	0	0	0	0	0.00
	1	0.0	0	0	0	0	0.00
	2	703.2	3015	12,614	23	15,652	22.26
	3	2562.3	13,042	54,571	90	67,703	26.42
Loblolly Pine—Atlantic White-cedar—Swamp Tupelo/Shining Fetterbush—Sweet-pepperbush Swamp Forest Association	0	91.7	141	0	0	141	1.54
	1	77.6	298	0	0	298	3.84
	2	88.5	543	0	0	543	6.14
	3	82.8	636	0	0	636	7.68
Loblolly Pine—Red Maple—Sweetgum/Switch Cane Ruderal Wet Forest Association	0	157.3	0	212	0	212	1.35
	1	14.5	0	0	0	0	0.00
	2	66.2	236	0	0	236	3.56
	3	19.8	0	0	0	0	0.00
Pond Pine—Loblolly-bay/Shining Fetterbush Swamp Woodland Association	0	219.0	321	0	0	321	1.47
	1	1144.1	4001	4026	688	8715	7.62
	2	3012.2	10,535	16,964	16,312	43,811	14.54
	3	4697.0	24,640	31,410	26,848	82,898	17.65
Saltmeadow Cordgrass—Saltgrass—(Black Needlerush) Salt Marsh Association	0	645.5	0	0	829	829	1.28
	1	383.2	0	0	1231	1231	3.21
	2	569.7	0	0	2929	2929	5.14
	3	129.9	0	0	835	835	6.43
Swamp Sawgrass Tidal Salt Marsh Association	0	93.1	0	0	202	202	2.17
	1	41.3	0	0	224	224	5.42
	2	74.5	0	0	647	647	8.68
	3	0.0	0	0	0	0	0.00
Inkberry—Shining Fetterbush—Honeycup Wet Shrubland Association	0	13.7	14	0	0	14	1.02
	1	44.1	118	494	0	612	13.88
	2	351.1	1506	6300	12	7818	22.27
	3	1987.4	10,116	42,324	70	52,510	26.42
Red Maple—Blackgum species—Sweetgum—Water Oak/Royal Fern species Swamp Forest Association	0	294.6	291	0	0	291	0.99
	1	68.4	108	0	0	108	1.58
	2	23.9	0	0	0	0	0.00
	3	2.4	0	0	0	0	0.00
Sweetgum—Red Maple—Swamp Tupelo/Cypress Swamp Sedge Swamp Forest Association	0	4.5	0	0	0	0	0.00
	1	34.4	0	0	0	0	0.00
	2	155.0	95	0	0	95	0.61
	3	20.1	0	0	0	0	0.00
Total area		17,955	69,656	1,68,983	50,940	2,89,579	214

^a^National Vegetation Classification Associations with a total area of less than 50 ha (Atlantic White-cedar/Swamp Bay/Shining Fetterbush—Large Gallberry Swamp Forest Association; European Common Reed Eastern Ruderal Marsh Association; Loblolly Pine—Red Maple—Sweetgum/Switch Cane Ruderal Wet Forest Association) and Developed Lands were excluded from C emissions estimation (total area excluded  =  367.5 ha)

^b^dNBR classes are: 0  =  unburned/very low severity (0–134), 1  =  low severity (135–168), 2  =  moderate severity (169–246), and 3  =  high severity (247–255)

Total mean below-ground C emissions were 5,237,521 t C, and ranged from 2,630,529 to 8,287,900 t C, as a function of the minimum and maximum of organic matter percent for each soil horizon within each soil series. The mean below-ground C emissions for the burn area was 1595.6 t C ha^−1^ and the below-ground C emissions range was 629.3–2511.3 t C sha^−1^ (Table 4). The C emissions were estimated for the seven soil series and their respective areas and burn depths within the fire perimeter. The USNVC vegetation association that had the highest below-ground C emissions corresponded to the communities with the highest above-ground C Emissions. The vegetation association with the highest soil C emissions were: the Pond Pine—Loblolly-bay/Shining Fetterbush Swamp Woodland Association (3,185,571 t C), the Pond Pine/Honeycup—Swamp Titi—Shining Fetterbush Wooded Wet Shrubland Association (1,000,190 t C), and the Inkberry—Shining Fetterbush—Honeycup Wet Shrubland Association (8,24,676 t C). The Black Needlerush Salt Marsh Association has the lowest soil C emissions (1087 t C), a result of the vegetation association’s characteristic standing tidal water on the soil surface across most of its spatial extent. The pine/hardwood swamp forest also had very low soil C emissions, caused by seasonally inundated standing water on the soil surface and high water table hydrology. The salt marsh vegetation class had intermediate soil C emissions with patchy spatial patterns associated with soil smoldering of deep tidal deposits of floating fine woody debris. The elevation gradient across the salt marsh vegetation class also contributed to drying of the surface soil horizons except during periods of high storm tides when the soils had ephemeral standing water.

**Table. 4:** Below-ground C emissions (t C) by National Vegetation Classification Associations

The US National Vegetation Classification Associations^a^	Area (ha)	C emissions range (t C)	C emissions mean (t C)	C emissions range (t C ha^−1^)	C emissions mean (t C ha^−1^)
Black Needlerush Salt Marsh Association	81.3	544–1631	1087	6.7–20.1	13.4
Pond Pine/Honeycup—Swamp Titi—Shining Fetterbush Wooded Wet Shrubland Association	3265.5	629,881–1,527,461	10,00,190	192.9–467.8	306.3
Loblolly Pine—Atlantic White-cedar—Swamp Tupelo/Shining Fetterbush—Coastal Sweet-pepperbush Swamp Forest Association	340.6	17,178–53,726	34,241	50.4–157.7	100.5
Loblolly Pine—Red Maple—Sweetgum/Switch Cane Ruderal Wet Forest Association	257.8	5,751–23,831	14,714	22.3–92.4	57.1
Pond Pine—Loblolly-bay/Shining Fetterbush Swamp Woodland Association	9072.3	1,447,770–5,110,122	3,185,571	159.6–563.3	351.1
Saltmeadow Cordgrass—Saltgrass—(Black Needlerush) Salt Marsh Association	1728.3	33,669–155,927	94,687	19.5–90.2	54.8
Swamp Sawgrass Tidal Salt Marsh Association	208.9	26,200–97,214	61,583	125.4–465.4	294.8
Inkberry—Shining Fetterbush—Honeycup Wet Shrubland Association	2396.3	460,597–1,286,446	824,676	20.6–536.8	344.1
Red Maple—Blackgum species—Sweetgum—Water Oak/Royal Fern species Swamp Forest Association	389.3	4710–17,788	11,236	12.1–45.7	28.9
Sweetgum—Red Maple—Swamp Tupelo/Cypress Swamp Sedge Swamp Forest Association	214.0	4229–15,385	9536	19.8–71.9	44.6
Total	17,955.1	2,630,529–8,287,900	5,237,521	629.3–2511.3	1595.6

^a^National Vegetation Classification Associations with a total area of less than 50 ha (Atlantic White-cedar/Swamp Bay/Shining Fetterbush—Large Gallberry Swamp Forest Association; European Common Reed Eastern Ruderal Marsh Association; Loblolly Pine—Red Maple—Sweetgum/Switch Cane Ruderal Wet Forest Association) and Developed Lands were excluded from C emissions estimation (total area excluded  =  367.5 ha)

## Discussion

### Comparison of C emissions from natural plant communities and their organic soils

Previous studies of C emissions from wildfires for forested peatlands in the coastal plain of eastern North Carolina have reported a range of C emissions from 0.2 to 11 kg C m^−2^ and total C emissions of 1–3.8 Tg C for four vegetation classes (hardwood forest, pine forest, shrub-scrub, and agriculture) [26], and 0.03–107.24 kg C m^−2^ and total C emissions of 9.47 Tg C for two ecological communities (woodland and shrubland) and agriculture crop vegetation [27]. The difference in the C emissions was attributed to burn depth, the first study based on a literature estimate of mean burn depths of 0.1–0.10 m [26] and the second study based on post-burn field measured mean burn depth of 0.42 m [27]. The range of C emission for the Pains Bay Fire in our study was 84.33–272.53 kg C m^−2^, and total emission of 5.5 Tg C. The mean post-burn field measured burn depth for the ten USNVC vegetation association communities (Table 4) was 0.13 m (ranging from 0.01 m in salt marsh communities to 0.25 m in pine woodland communities).

The Pond Pine—Loblolly-bay/Shining Fetterbush Swamp Woodland Association (9072.2 ha), Pond pine/Honeycup—Swamp Titi—Shining Fetterbush Wooded Wet Shrubland Association (3265.5 ha), and the Inkberry—Shinning Fetterbush—Honeycup Wet Shrubland Association (2396.3 ha) comprised 80% of the total land area within the fire perimeter and 82% of the total wildfire C emissions. Ecological factors that contribute to vegetation patterns in these USNVC Associations are controlled by three ecological gradients: soil pH, nutrient availability, and depth to the water table. The three vegetation communities are all encompassed within the Pungo soil series and the USDA Soil Conservation Service typical pedon of Pungo muck soil is located within the study’s wildfire perimeter. The Pungo soil pH is 2.0–4.4 to a soil depth of 1.8 m, nutrient availability is characterized as very poor and only suitable for woodland management, and the depth to the high water table is 0–0.3 m during the months of November–May [57]. The vegetation gradient from woodland to shrubland in these three vegetation associations is likely attributable to the reduction in the water table depth across the elevation gradient of 3.6–20 m, the reduction in the temporal duration of the high water table with increasing elevation, and the reduction in surface water flow rates with increasing elevation. All three vegetation associations had a similar reduction in post-burn soil elevation from combustion of the organic soil Oe and Oa horizons (0.21–0.25 m), indicating the high water table was likely similar across the vegetation gradient at the time of the wildfire smoldering stage and the soil moisture content at a depth below 0.25 m was greater than 260 percent. Reardon et al. [58] previously sampled peat and muck soil series from within the study area in order to determine the relationship of soil moisture, mineral content, and ignition and smoldering potential. Muck soil series were found to smolder at higher moisture contents than loamy root mat soils. Muck soil with a moisture content of 201% had an estimated 50% probability of sustained smoldering. At soil moisture contents above 260% the estimated probability was less than 10%. Sustainable smoldering combustion decrease by 17.2% with each 5% increase in soil moisture content. The increase in organic soil combustion and reduction in soil elevation can likely be attributed to the increased radiation heating from the combustion of larger quantities of above-ground fuel loads in the woodland vegetation classes versus shrub classes.

Poulter et al. [26] reported that a similar North Carolina peatland wildfire had no correlation between peat burn depths, increasing higher fire severity, and lower surface elevations. Their analysis of elevation cross sections for the burn area also showed no relationship between fire severity and surface elevation. The higher-severity fire was more commonly associated with peat depths and high fuel loads, but did not correlate with depressions in the peat surface profile.

**Fig. 5:**
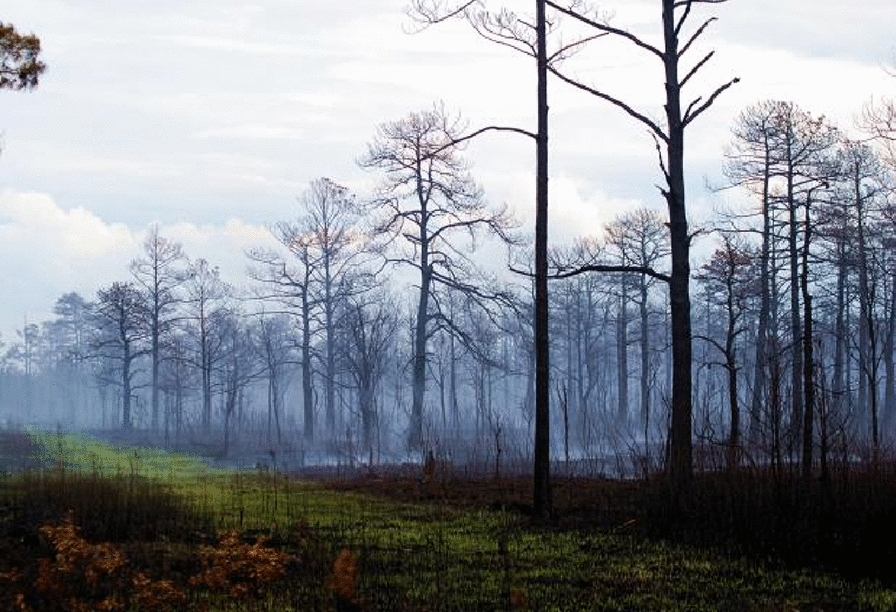
Regreening and subsequent horizontal flaming and smoldering of herbaceous vegetation and surface organic soil following the wildfire flaming crown fire stage

The Pains Bay Fire peat burn depths and high ground fire frequency classes were associated with historical commercial forest land management practices that included artificial drainage and ditching to lower the water table and reduce surface soil moisture content for tree plantations. The proximity of plantation v-ditches to larger road ditches which drain into the Pamlico Sound facilitated the lowering of the surrounding surface water table and soil moisture content. Areas with low ground fire frequency classes exhibited the smoldering process described by Reardon et al. [58] in which thin layers of organic soil were combusted to the soil depth where soil moisture content exceeded 260%, followed by a sun and wind drying interval of day and a subsequent horizontal and vertical soil surface layer flaming and smoldering. This repeating horizontal smoldering, extinguishing, soil surface drying, and horizontal smoldering cycle resulted in several layers of the organic material ignition over the duration of the wildfire, frequently of sufficient duration to allow for the regreening of fern species and their subsequent combustion (Fig. 5).

In addition to C emission, peatland wildfires release non-CO_2_ gas emissions during the flaming and smoldering stages. Although the focus of this study was on the C emissions, aldehydes and volatile organic compounds (VOCs) samples were collected using US Environmental Protection Agency protocols (TO-15 and TO-11A/8315 HPLC) during the smoldering stage of the fire. The highest VOC emissions [ranging from 74.56 to 10.86 parts per billion by volume (ppbv)] were identified as acetone, benzene, toluene, chloromethane, hexane, and heptane. Aldehyde emissions ranged from 0.404 to 0.100 µg per liter (µg/l) for acetaldehyde, formaldehyde, propionaldehyde, isovaleradehyde, o-tolualdehyde, and valeradelhyde.

### Fire severity in natural plant associations

The vegetation distribution in natural plant associations in North America temperate peatlands is controlled by soil pH, nutrient availability, and depth to the water table [30]. The heterogeneity of vegetation is a product of the spatial and temporal patterns of the water table level and the surface wetness. In temperate peatlands, the natural floristic pattern can be typically characterized as containing shrub species in the central portions of the peatlands and gradations to the margins that favor trees and tall shrubs, and salt marshes at the shorelines. The majority of temperate peatlands in Southern North America are dissected by roads and adjacent ditches, and damming structures that disrupt the natural elevation gradient of the peatland and favor the establishment of areas of atypically higher and lower water tables [33]. In response to these man-made disturbances, vegetation assemblages become increasingly heterogeneous, and in many instances, the sudden disturbance favor the establishment of nonnative and invasive species [i.e., *Phragites australis* (Cav.) Trin. ex Steud. (European common reed)].

Within the Pains Bay Fire perimeter, fire severity of natural plant associations was determined by the proximity of the water table to the soil surface, the biomass of litter and fine and course woody debris, and the live fuel moisture status of shrubs and trees. Figures 1, 2 illustrate the extreme fire severity effects that were associated with shrubland and pine woodlands. During the first week of the flaming stage of the fire, the pond pine woodlands exhibited a running crown fire that consumed 100% of the tree and shrub foliage and cones, and heat killed the tree and shrub canopies and bole to the soil surface. Figure 5 illustrates the extent of the tree and shrub canopy consumption. During this phase of the fire, organic soil was consumed to the depth where soil moisture content was  >  260%. During the subsequent smoldering stage of the fire, the soil surface drying from sun and winds reduced the soil moisture content to  <  260% and the soil surface reignited with a series of flaming stage soil combustion events that occurred over several months. Figure 3 illustrates the extended duration of organic soil consumption that resulted from the lowering of the water table from historical ditching by forest industry to enhance the productivity of pine plantations.

## Conclusions

The mean peat burn depth of the Pains Bay Fire peatland wildfire (18,329 ha) was 0.13 m. Total above-ground C emissions were 0.26 Tg C within the burn area perimeter. Total mean below-ground C emissions were 4.75 Tg C, for total fire emissions of 5.01 Tg C. In contrast, the Evans Road Fire peatland wildfire (16,814.2 ha), located 60 km to the east, had a mean peat burn depth of 0.42 m and resulted in estimated below-ground fire emissions of 9.16 Tg C and above-ground fire emissions of 0.31 Tg C, for total fire emissions of 9.47 Tg C. The Evans Road Fire emissions [27] are comparable to the Pocosin Lakes Wildfire [26] which reported a lower maximum burn depth of 0.10 m. Although all three wildfires occurred on temperate peatlands in adjacent counties on the coastal plain of North Carolina, the Evans Road Fire was comprised of a large area with water control structures that artificially lowered the surface water table and resulted in reduced soil moisture and a tripling of the depth of organic soil consumption and a near doubling of the C emissions for a relatively similar burn perimeter area. These wildfires demonstrate the importance of the proximity of the water table to the soil surface, maintaining soil moisture content  >  260%, and maintaining the live fuel moisture status of shrubs and trees  >  150%, in order to reduce short-term flaming and long-term smoldering phase combustion.

These studies demonstrate that in contrast to undisturbed temperate peatlands, human induced disturbances of the natural elevation gradient of the peatland has resulted in increased heterogeneity of floristic variation and assemblages that are a product of the spatial and temporal patterns of the water table level and the surface wetness across peatlands. Human induced changes in surface hydrology and land use influenced the fuel characteristics of natural vegetation and associated soils, thus influencing wildfire behavior and resulting C emissions.
